# Successful and Safe Long-Term Standard Antiviral Therapy in a Patient with “*Explosive”* Immune Response in Course of HCV-Related Liver Cirrhosis

**DOI:** 10.3390/ijms160614075

**Published:** 2015-06-19

**Authors:** Paolo Conca, Giovanni Cafaro, Amalia De Renzo, Antonio Coppola, Ernesto Cimino, Giovanni Tarantino

**Affiliations:** 1 Department of Clinical Medicine and Surgery, Federico II University Medical School of Naples, 80131 Naples, Italy; E-Mails: paolo.conca@unina.it (P.C.); giovanni.cafaro@hotmail.it (G.C.); antocopp@unina.it (A.C.); ernesto.cimino@unina.it (E.C.); 2 Haematology Unit, Federico II University Medical School of Naples, 80131 Naples, Italy; E-Mail: amalia.derenzo@unina.it

**Keywords:** hepatitis C infection, lymphoproliferative disorders, cryoblobulins, rheumatoid factor, antiviral treatment

## Abstract

Hepatitis C virus (HCV) has been recognized to be both a hepato- and lymphotropic virus. HCV lymphotropism represents an essential detail in the pathogenesis of virus-related autoimmune and lymphoproliferative disorders, ranging from clonal expansion of B-cells with organ and non-organ-specific autoantibody production up to overt non-Hodgkin’s lymphoma along a continuous step-by-step model of B-cell lymphomagenesis, where the intermediated mixed cryoglobulinemia could be considered as a stage of suppressible antigen-driven lymphoproliferation. The HCV long-lasting extrahepatic replicative state generates an abnormal systemic immunological response, including rheumatoid factor (RF) and cryo- and non-cryoprecipitable immune complexes, as well as clinical manifestations, comprising dermatitis, polyarthralgias and arthritis, pulmonary disease, aplastic anemia, glomerulonephritis and vasculitis. The mechanism of these extra-hepatic disorders is thought of as linked to immune complex disease, but their pathogenesis is poorly clarified. Immune-suppressive treatment could induce high-level hepatitis C viremia and impair hepatic disease. We report a female patient, whose chronic HCV-related liver cirrhosis with associated explosive, but oligosymptomatic lymphoproliferative immune response, *i.e.*, RF beyond three thousand times the upper of normal range (unr), type II cryoglobulinemia with cryocrit 40% and monoclonal gammopathy IgM-*k*, has been successfully and safely treated by long-lasting (sixty-six months) combined antiviral therapy (pegylated interferon alfa and ribavirin), at moderate and tapering dose regimen, prolonged for nearly 24 months after the first viral suppression. At the last follow-up (fifty-one months), the patient was showing very-long term antiviral response, progressive decline of secondary immune activation and absence of significant side-effects. Further research is required to fully verify the real impact on therapeutic choice/regimen.

## 1. Introduction

Interaction between Hepatitis C virus (HCV) and the immune system represents a well-known pathogenic mechanism that causes liver and extrahepatic damage in chronically infected patients. Several reports in the literature have confirmed that HCV infection leads to different types of immune disease such as cryoglobulinemia, rheumatoid arthritis, Sjogren’s syndrome, hemolytic anaemia and severe thrombocytopenia, autoimmune hepatitis, thyroid disorders and diabetes mellitus. These manifestations, labelled as autoimmune diseases, are reckoned to be caused by the lymphotropism of the virus: particularly, the interaction between the E2 glycoprotein of the HCV envelope and the CD81 receptor, expressed by hepatocytes and B lymphocytes, could play a fundamental role. Chronically infected lymphocytes constitute a reservoir of virus, but on the other hand persistent infection of lymphoid tissue induces a chronic stimulation of the immune system based on various molecular mechanisms, of which the activation of Bcl-2, a proto-oncogene that prolongs lymphocytes survival, and induces a switch of T lymphocytes both in Th2 phenotype and Th1 phenotype, plays a key role. The Th2 phenotype increases humoral immunity and production of autoantibodies, while the Th1 phenotype causes mixed cryoglobulinemia (MC) and immune disorders affecting various organs [1]. HCV chronic infection is the main cause of MC and is observed in 40%–90% of affected patients. Cryoglobulins are immunoglobulins exerting a rheumatoid factor (RF) activity and creating circulating immune-complexes that precipitate in different tissues and cause several clinical manifestations [2]. Moreover, patients with MC present higher risk of HCV related non-Hodgkin lymphomas (HCV-NHL). MC could be considered an intermediate stage leading to NHL in this subset of patients. To explain this correlation two hypotheses have been taken into consideration: The theory of the signal of proliferation and the increased expression of activation-induced cytidine deaminase (AICD). Together with signal of proliferation, HCV could increase mutation rate by inducing AICD expression [3]. In 2012 prevalence of HCV infection in patients with B-NHL was nearly 15%, while a 2.5 relative risk (RR) (95% confidence interval, CI, 2.1–3.1) in case-control studies and 2.0 RR (95% CI, 1.8–2.2) in cohort studies were reported. In the afore-mentioned reports, infection persisted for many years before NHL was diagnosed. Studies have suggested a stronger association with lymphoplasmacytic/Waldenstrom (LP/W) lymphomas [4]. A more recent study has showed that marginal zone lymphoma (MZL) and large B cells diffuse lymphoma (DLCBL) are the most frequent histo-types in chronically HCV infected patients [5]. In this setting, DLCBL is less dependent on HCV infection and less responsive to antiviral therapy (AT), and for this reason, some do not consider AT representing the first line treatment of this disease. Of interest, HCV patients show, more frequently than the general population, a clonal B-cell proliferation without a better-defined lymphocytosis [5]. Here, we present the case of a woman with notable alterations of immune laboratory data, related to HCV infection, changing during AT administration.

## 2. Case Report

On December 2004 a 61 year-old female patient affected by HCV-related liver cirrhosis was observed, and manifested the first and unique episode of hydro-saline retention, evidenced as poorly responsive to diuretic therapy, non-tense, ascites and pre-tibial palpable oedema with a minimal pericardial effusion (Child-Pugh score B8; MELD 11). Medical history revealed anti-HCV antibodies, which were documented from 1991, and a dysmetabolic phenotype characterized by visceral obesity, HDL cholesterol reduction, impaired fasting glucose and arterial hypertension controlled by drug therapy; laboratory parameters and imaging features evidenced initial organ damage (basal micro-albuminuria in a 24 hour-collected sample, bilateral carotid atherosclerosis and left ventricular hypertrophy with Doppler ultrasound). Therefore, she was admitted to our ward and was provided with a full clinical laboratory and instrumental evaluation. The virological markers of HCV infection showed infection by HCV, 1b genotype, moderate plasmatic levels of HCV-RNA (230,000 UI/mL) and C/T polymorphism of IL 28B gene promoter. Markers of other hepatotropic viruses, *i.e.*, cytomegalovirus, Epstein-Barr virus and herpes simplex virus and autoimmunity (ANA/AMA/anti LKM as well as serum anti-gliadin and anti-endomysium) were negative. Hemocromocytometric analysis showed moderate anemia (RBC 3,170,000; HB 9.5 g/dL) and low platelets count (PLT 97,000/mm^3^). The patient presented with abnormal levels of laboratory parameters, suggesting the presence of cytolysis and cholestasis, *i.e.*, AST 265 U/L (normal value, nv < 35), ALT 241 U/L (nv < 35), ALP 108 U/L (nv < 104), gammaGT 67 U/L (nv < 36), direct bilirubin 0.42 mg/dL (nv < 0.3) LDH 468 U/L (nv < 450), as well as of impaired liver synthesis; she showed hypo-albuminemia (2.7 g/dL albumin concentration) and typical alterations of coagulation suggesting a subacute-chronic disseminated intravascular coagulopathy (DIC) (PT INR: 1.49 (nv < 1.3), anti-thrombin III: 44.7% (nv 70%–120%), d-dimerus: >6500 μg/L (nv < 198), fibrinogen degradation products (FDP): 20–40 (nv < 10). Markers of liver proliferation were raised (alpha-fetoprotein, AFP: 24.7 ng/mL (nv < 15), tissue polypeptide specific antigen, TPS: 6717 ng/mL (nv < 80)). Imaging features (US and CT of abdomen) evidenced slight increase of the liver volume (mainly at level of the right lobe), hypertrophy of the caudate and left lobes, irregular edges, coarse pattern, splenomegaly (longitudinal diameter, LD: 165 mm) and ascites. Gastroscopy evidenced signs of portal hypertension (congestive gastropathy). The ascitic episode was resolved after three weeks by infusion of plasma-expanders (albumin 20 g 3 times/week) and combined diuretic therapy (furosemide 40 mg *i.v*. 3 times/week + potassium kanreonate 200 mg/day *per os*). Moreover, a marked immune activation represented by the following data was noted: typization of cryoprecipitate: IgM-*k*, II type, mixed-, mono- and poly-clonal; cryocrit: 40%, RF: 53,200 UI/mL (nv ≤ 15; 3546 × unr), monoclonal component IgM: 0.61 g/dL, k type, IgM total: 37.6 g/L (nv 0.4–2.3), IgG total: 25.9 g/L (nv 7–16), circulating immune complexes (CIC) C1q: 86 mcg/mL (nv < 40), CIC C3d: 62 mcg/mL (nv < 24), anti-cardiolipine antibodies: 40.5 U/mL (nv < 20) and beta2-microglobulin: 5203 ng/mL (nv < 2740) (Table 1). The erythrocyte sedimentation rate (ESR) was 88 mm (1 h nv < 10), At clinical examination, findings related to the presence of cryoglobulinemia, such as skin discromies, sign of recent or past cutaneous vasculitis, nor superficial enlarged lymph nodes, fever, pruritus, weight loss, night sweats, and asthenia were not present. Immune alterations prompted us to perform bone marrow biopsy and hematological evaluation. Bone marrow biopsy showed, in a context of a normal tri-linear population of hematopoietic precursors, some nodular aggregates of small abnormal lymphoid cells in association with lympho-plasmacellular elements. Reticulin was found slightly thick around infiltrates. These histology features suggested a lymphoproliferative disease characterized by small lymphoid cells. In accord with pathology and laboratory findings the hematologist diagnosed an IgM-gammopathy associated with HCV-related cryoglobulinemia. Therefore, the patient underwent a close follow-up based on a three-month-control of the monoclonal component and on performing imaging detection of deep and superficial lymph nodes to track an evolution into a lympho-plasmacytic/lymphoplasmacytoid lymphoma.

**Table 1:** Principal clinical, laboratory and instrumental data at baseline.

Overall Abnormal Findings
Mild-moderate ascites (Child-Pugh score B8 (Alb 3, ascites 2; MELD 11)). Dysmetabolic phenotype characterized by visceral obesity, hepatic steatosis, HDL cholesterol reduction, impaired fasting glucose and arterial hypertension. Organ damage characterized by basal microproteinuria in the 24 hour-collected sample, bilateral carotids atherosclerosis and left ventricular hypertrophy at ultrasound. High markers of hepatic cytolysis and cholestasis (AST 265 U/L (nv < 35), ALT 241 U/L (nv < 35), ALP 108 U/L (nv < 104), gamma-GT 67 U/L (nv < 36), direct bilirubin 0.42 mg/dL (nv < 0.3) LDH 468 U/L (nv < 450)). Subacute-chronic disseminated intravascular coagulopathy (DIC) (PT INR: 1.49 (nv < 1.3), Anti-thrombin III: 44.7% (nv 70%–120%), d-dimerus: 6500 μg/L (nv < 198), FDP (fibrinogen degradation products): 20–40 (nv < 10)). Impaired markers of liver proliferation (AFP: 24.7 ng/mL (nv < 15), TPS: 6717 ng/mL (nv < 80)). Marked immune activation (typization of cryoprecipitate: IgM-*k*, II type, mixed-, mono- and poly-clonal; cryocrit: 40%), rheumatoid factor: 53,200 UI/mL (nv ≤ 15; 3546 × unr), monoclonal component IgM: 0.61 g/dL, *k* type, IgM total: 37.6 g/L (nv 0.4–2.3), IgG total: 25.9 g/L (nv 7–16), ESR: 88 mm (nv at 1 h < 10), CIC C1q: 86 mcg/mL (nv < 40), CIC C3d: 62 mcg/mL (nv < 24), Ab anti-cardiolipine: 40.5 U/mL (nv < 20), beta2-microglobulin: 5203 ng/mL (nv < 2740)). Histology features suggesting a lymphoproliferative disease characterized by small lymphoid cells at bone marrow biopsy.

On January 2005, the patient started only pegylated IFN alfa 2b 1.5 mcg/kg/week. The choice was due to both reduced Hb levels and Glomerular Filtration Rate (GFR), detected during routine check-ups in the course of diuretic therapy, adding Ribavirin (RBV) after six months only.

The aim of AT was the suppression of the viral antigenic driver and the reduction of virus-related immune stimulation.

On May 2005 (the fifth month of monotherapy with IFN alpha): The patient manifested a transitory purpuric rash on legs that spontaneously resolved after 10–14 days. Recurrence of rash was not observed. Slight dyschromia, which was in accordance with a histological pattern of “mixed cytoclastic dermatitis”, persisted.

On June 2005 (the sixth month of AT): The patient continued the administration of pegylated IFN alfa 1.5 mcg/kg/week and received a 800 mg-dose of RBV, with Hb and GFR levels ameliorated.

Peripheral oedemas and ascites resolved. Immune modulating and/or cyto-reductive therapies such as rituximab/corticosteroids at high dosage/cyclophosphamide/plasmapheresis were considered as alternatives in case of failure, but they were not thought to be necessary. Of interest, only in this period did the patient reach the greatest peak of RF 58,200 UI/mL (nv ≤ 15; 3880 × unr) and cryocrit (60%); at same time the first and unique episode of consumption of complement was observed: C4: 0.081 g/L (nv 0.1–0.4). IgM: 23.8 g/L (nv 0.4–2.3; trend of reduction) (Table 2a). Moreover, the patient had a decrease in viral plasma levels (quantitative HCV-RNA: 95,000 UI/mL with a reduction of one log^10^), improvement of anaemia (RBC 3,890,000; Hb 11.5 g/dL) and of platelets count (PLT 72,000/mm^3^) and other laboratory liver tests.

On March 2006 (the 15th month of therapy and the ninth month of RBV combined therapy) the patient showed reduction of immune activation parameters (RF: 35,000 UI/mL (nv ≤ 15; 2333 × unr), cryocrit: 35%, IgM: 18.8 g/L (nv 0.4–2.3), IgM-*k* monoclonal component (MC): 0.58 g/dL, beta2-microglobulin: 4700 ng/mL (nv < 2740), first normalization of liver cytolysis and residual plasmatic levels of virus (8700 UI/mL with a reduction of two log^10^)) (Table 2a); however, the Positron Emission Tomography imaging of total body evidenced abdominal lymphadenopathy without areas of pathologic increase of uptake in association with reactive axillary and inguinal small multiple lymph nodes (in the sub-centimetric order). Residual plasma levels of virus, the polydistrectual lymphadenopathy presence and the persistence of markers of immune stimulation, all needed a prolongation of AT, which was accompanied by a close follow-up. Because of a favourable profile of tolerability, both support therapy for eventual side-effects, and growth factor administration were not necessary.

On September 2006 (the 21st month of therapy, and the 15th month of RBV combined therapy) a further reduction of immune activity was observed, RF: 30,000 UI/mL (nv ≤ 15; 2000 × unr), cryocrit: 32%, IgM: 15.3 g/L (nv 0.4–2.3), IgM-*k* MC: 0.58 g/dL, beta2-microglobulin: 4730 ng/mL (nv < 2740); again, normalization of liver cytolysis and reduction of plasmatic levels of virus were confirmed (1200 UI/mL, *i.e.*, reduction of two log^10^); moreover, sentinel superficial lymph nodes disappeared and the patient had the opportunity to continue AT.

Between March 2007 (the 27th month of therapy and the 21st month of combination with RBV) and September 2007 (the 33rd of therapy and the 27th month of combination with RBV): A further reduction of immune activation was observed (RF: 17,000 UI/mL (nv ≤ 15), cryocrit: 30%, IgM: 9.7 g/L (nv 0.4–2.3), IgM-*k* MC: 0.74 g/dL, beta2-microglobulin: 4890 ng/mL (nv < 2740), (Table 2a); both normalization of parameters of liver cytolysis and minimal plasmatic levels of virus (a reduction of four log^10^) persisted. In addition to the resolution of sentinel superficial lymphadenopathy, the patient experienced a regression of deep abdominal lymph nodes, while isolated enlarged lymph nodes at the hepatic hylus persisted (maximum diameter of 13 mm at abdominal CT); however, during this period a moderate intolerance to AT occurred and a reduction of therapeutic dosage was needed; therefore, the patient switched to pegylated-IFN alfa 2b, 1 mcg/kg/week, and RBV, 600 mg/day.

On March 2008 (the 39th month of AT and the 33rd month of combined therapy, IFN plus RBV)—the 12th month of reduced dosage: In association with reduced immune activation and confirmed normalization of liver cytolysis (RF: 15,000 UI/mL (nv ≤ 15), cryocrit: 27%, IgM: 5.7 g/L (nv 0.4–2.3), IgM-*k* MC: 0.62 g/dL, beta2-microglobulin: 4420 ng/mL (nv < 2740)), the patient experienced first virological remission and viral plasma levels were undetectable using PCR Real Time.

Between March 2009 (the 51st month of therapy and the 45th month of combination with RBV—the 24th month of reduced dosage—12 months of virological remission) and March 2010 (the 63rd month of therapy and the 57th month of combination with RBV—the 36th of reduced dosage—the 24th month after the first virological remission) the patient was showing normal laboratory liver tests and improved immunological and virological parmameters: (RF: 4300 UI/mL (nv ≤ 15), cryocrit: 20%, IgM-*k* MC: 0.57 g/dL, beta2-microglobulin: 3620 ng/mL (nv < 2740), absent HCV viremia (RealTime)), (Table 2). Virological and immunological remission permitted further reduction of the therapeutic dosage and the patient switched to 0.75 mcg/kg/week of pegylated IFN alfa 2b and 400 mg/day of RBV.

On June 2010 (the 66th month of therapy and the 60th month of combination with RBV—the 39th month of reduced dosage and three months after the switch to 0.75 mcg/Kg/week and 400 mg/day of RBV)—27 months after first virological remission, the patient ended AT. Main laboratory examinations confirmed both virological/immunological remission and normalization of liver tests: absent plasma viral levels (RealTime) indicating *End Therapy Response*), RF: 2780 UI/mL (nv ≤ 15), cryocrit: 19%, IgM-*k* MC: 0.54 g/dL, beta2-microglobulin: 3120 ng/mL (nv < 2740) (Table 2). Only multiple small and subcentimetric lymphadenopathies at axilla and inguinal region and splenomegaly (with a LD of 150 mm at ultrasonography of abdomen) persisted.

Of particular note, ultrasonographic examination revealed axillary and inguinal lymph nodes exhibiting tapered shapes and a hyperhecoic hylus (typical feature of reactive lymphadenopathies).

Between June 2010 and October 2014 the patient underwent a prolonged and well-documented follow-up that confirmed both normal laboratory liver parameters—AST: 19 U/L (nv < 35), ALT: 15 U/L (nv < 35), total proteins: 8.0 g/dL (nv 6.5–8.2), albumin: 4.5 g/dL, total cholesterol: 187 mg/dL, LDH: 403 U/L (nv < 450), AFP: 1.8 ng/mL (nv < 15), TPS: 75 ng/mL (nv < 80) and virological remission with absent plasma levels of virus at 51st month of follow-up indicating a *very Long-Term Response*; a minimal immune activity—RF: 1560 UI/mL (nv ≤ 15, *i.e.*, 104 × unr), cryocrit: 12% , IgM: 5.07 g/L (nv 0.4–2.3), CIC C1Q: 71 μg/mL (nv 0–40), CIC C3d: 116 μg/mL (nv 0–24), IgM-*k* MC: 0.77 g/dL, beta2-microglobulin: 3115 ng/mL (nv < 2740), (Table 2b). The antibody anticitrulline test, only performed on December 2010, resulted negative. A moderate spleen enlargement persisted, *i.e.*, LD of spleen 142 mm. Interestingly, bone marrow showed normalization of previous features: reduction of nodal aggregates, thin reticulin, absence of atypical cells and marked decrease of the plasmacellular population. Also the immune phenotype did not show alterations of B- and T-lymphocytes. Finally, cytogenetic analysis of the bone marrow excluded the t(14; 18) (q32; q21) translocation and a possible evolution to a centro-follicular lymphoma.

Table 2(**a**) Laboratory parameters during the antiviral therapy; (**b**) Laboratory parameters during the follow-up.

**Table:** (**a**)

Parameters	The 6th Month of Therapy	The 15th Month of Therapy	The 33rd Month of Therapy	The 51st Month of Therapy	The 66th Month of Therapy (End of Therapy)
**Alanine aminotransferase (U/L) nv < 35**	78	32 ^b^	27	30	27
**Cryocrit (%)**	60	35	30	23	19
**Rheumatoid Factor (IU/mL) nv ≤ 15**	58,200 ^a^	35,000	17,000	11,000	2780
**IgM-*k* monoclonal component (g/dL)**	Not measured	0.58	0.74	0.59	0.54
**IgM levels (g/L) nv 0.4–2.3**	23.8	18.8	9.7	2.2	2.0
**Beta 2-microglobulin (ng/mL) nv < 2740**	4300	4700	4890	4120	3120
**HCV—RNA (IU/mL) Cut-off < 12**	95,000	8700	Minimal viremia	Absent	Absent

**Table:** (**b**)

Parameters	The 6th Month of Follow-up (SR) °	The 18th Month of Follow-up (LTR) °°	The 51st Month of Follow-up (vLTR) °°°
**Alanine aminotransferase (U/L) nv < 35**	27	27	15
**Cryocrit (%)**	18	16	12
**Rheumatoid Factor (IU/mL) nv ≤ 15**	1280	472	1560
**IgM-*k* monoclonal component (g/dL)**	0.64	0.70	0.77
**IgM levels (g/L) nv 0.4–2.3**	2.0	1.7	5.07
**Beta 2-microglobulin (ng/mL) nv < 2740**	2640	2637	3115
**HCV—RNA (IU/mL) Cut-off < 12**	Absent	Absent	Absent

^a^: The highest value of the rheumatoid factor; ^b^: First recovery of liver cytolysis; ° SR: Sustained Response; °° LTR: Long-Term Response; °°° vLTR: very Long-Term Response.

## 3. Discussion

The patient presented with a dysregulated IgM gammopathy showing humoral (RF) and cryoprecipitate (mixed cryoglobulinemia of type II) activity induced by an amplified HCV replication. Gammopathy could be not classified as Waldenstrom macroglobulinemia or nodular lymphoid hyperplasia (NLH), which is a lymphoproliferative disease with small cells due to a possible lymphoplasmacytic/lymphoplasmacitoid immunocytoma. These observations are in accordance with previously mentioned studies, which revealed a higher incidence of immune stimulation and lymphocytosis in HCV patients; particularly, infection can be an antigenic trigger of lymphocytic stimulation and persist for several years before NLH occurrence. Therefore, lymphocytic stimulation can be considered an important factor to predict evolution to lymphoma [4,5]. Of major regard, the patient had an IgM hypergammaglobulinemia of undetermined significance (MGUS), which represents an important marker present in many chronically infected patients [6]. The prolonged AT led to reduction of HCV-RNA levels and reduction of cryoglobulinemia and MGUS. Beneficial effects of AT on lymphoid proliferation have been already established by several trials. A prospective cohort study, evaluating the efficacy of new combined therapies with Peg-IFN alpha/RBV/first generation-prothease inhibitors (Boceprevir and Telaprevir) in patients with vasculitis due to HCV-related mixed cryoglobulinemia (MC-HCV) showed that patients, who presented with virological remission, had a reduction of cryoglobulins levels and recovery of C4 fraction of the complement [7]. A multi-centric Italian study evidenced that AT may be useful to reduce the immune activation in MC-HCV patients having received treatment both for a seven month- and 12 month-period. The majority of responder patients had an attenuation of purpura and arthralgias and evidenced a complete and persistent resolution of symptoms. However, many patients had a symptomatic relapse after the AT treatment was stopped [8]. A recent meta-analysis confirmed efficacy and safety of therapy in MC-HCV patients [9]. Hermine *et al*. evidenced remission of splenic lymphoma with hairy lymphocytes related to HCV infection: of interest, partial and complete remission of NLH were characterized by the absence of detection of serum HCV-RNA, while in one patient recurrence of HCV-RNA led to relapse of lymphoma [10]. The Hermine study was the first trial demonstrating the direct correlation between HCV and NLH and the possibility to cure lymphoma by eradicating HCV infection. Another multicentric Italian trial evaluated the impact of AT in course of indolent B-NLH-HCV; the hematologic response was significantly associated with the clearance or reduction of viral load induced by AT and was more frequent in genotype 2 carriers [11]. On the contrary, in a further trial patients not presenting sustained virological response (SVR) showed positive markers of lymphoproliferation and neutralizing antibodies direct against E1- and E2-HCV antigens. Instead, obtaining SVR was inversely related to positivity of these tests [12]. Moreover, in our patient immune activation did not determine severe symptoms: only a transitory and spontaneously resolving purpuric rash at legs was noted; the patient showed a rash contextually to the peak of the immune activation and after a five month-course of IFN therapy. Also an uncertain, and mild kidney damage, type glomerulonephritis, was described; however, this manifestation might be caused by long-lasting hypertension, which was poorly controlled during the follow-up. Because of alteration of the kidney function and reduction of Hb levels, we performed an IFN monotherapy during the first five months and asynchronous combination with RBV from sixth month of AT. In light of the minimal pathologic phenotype, we preferred an aetiologic therapeutical approach (without plasmapheresis or cytotoxic/immune modulating therapy with rituximab/corticosteroids at high doses/cyclophosphamide) in order to modulate the antigenic viral trigger (pegylated IFN alfa 2b + RBV). Supporting our choice, there is the evidence that AT could play a concomitant important role to treat MC-HCV patients on Rituximab (RTX). A cohort prospective study evidenced that AT, together with RTX, improved renal function and cryoglobulins clearance. Patients who had benefit from RTX/AT combined therapy obtained a more rapid clinical remission. AT was tolerated and inhibited viral rebound during RTX treatment [13]. Similarly, AT could play a fundamental role in previously treated patients with chemotherapy or immune therapy. La Mura *et al.* studied AT efficacy in patients who were underwent chemotherapy to cure aggressive forms of HCV-non-Hodgkin lymphomas (NHLs); patients with SVR had no relapse of NHLs and responders were characterized by longer survival time without disease [14]. AT represents a rational therapeutic choice to treat HCV-NHLs: in indolent HCV-NHLs, AT is the first line therapy, with both an early chemotherapy, or immune therapy not indicated [15]. In low-grade HCV-NHLs, AT can be used alone or together with chemotherapy. Instead, aggressive variants need chemotherapy as an initial therapy, because AT is not sufficient to ameliorate the disease progression. However, as suggestive rule, after patients complete chemotherapy cycles or obtain clinical remission, AT could remove a potential trigger for NHLs relapse [15,16]; consequently, in moderate or high grade HCV-HNLs, AT can prolong the disease-free time [6]. On the other hand, lymph tropism and bone marrow tropism of HCV could compromise complete viral clearance and negatively influence liver disease. Chronically infected B-cells, in particular some long-life subpopulations, represent important reservoirs favouring the relapse of HCV infection, also in patients who obtain SVR [16].

## 4. Future Directions

AT was an efficient approach in our patient affected by HCV-related liver cirrhosis, improving laboratory parameters. A fundamental element of HCV chronic infection is the abnormal immune response against viral antigens that frequently leads to a permanent chronic immune reaction. Although this response, well modulated, is central to the virus eradication, the same response could cause liver damage and provoke a dysregulated immune activation leading to severe clinical manifestations, such as MC or NHLs. Particularly, chronic lymphocytic proliferation and hypergammaglobulinemia in some conditions evolve to lymphoproliferative syndromes, which could manifest without severe symptoms, at least at an initial stage. Therefore, in addition with evaluation of laboratory liver tests, immune activation needs careful consideration to decide an optimal and safe therapeutic strategy, *i.e.*, combined AT and evaluation of the long-term prognosis. Thus, in these patients we suggest that physicians carefully evaluate some markers of immune activation, such as cryoglobulins, RF, fractions of complement, circulating immunocomplexes, presence of mono-clonal gammopathy or poly-clonal hypergammaglobulinemia and typical symptoms of MC. In fact, these parameters could indicate an urgent necessity to consolidate therapy, obviously in selected patients, in order to eradicate the latent infection of B-lymphocytes and reduce both the incidence of MC/NHL and relapse of liver disease. As a final observation, the content of this case report prompts a revision of the optimal timing of the newly appearing direct-acting, IFN-free antiviral combinations.

## 5. Conclusions

At the last follow-up (fifty one months), the patient was showing very-long term antiviral response, progressive decline of secondary immune activation and absence of significant side-effects. Further research is required to fully verify the true impact of therapeutic choice/regimen in similar cases.
